# A *de novo* Non-sense Nuclear Factor I B Mutation (p.Tyr290*) Is Responsible for Brain Malformation and Lung Lobulation Defects

**DOI:** 10.3389/fped.2022.865181

**Published:** 2022-03-30

**Authors:** Hao Huang, Jieyuan Jin, Liping Wu, Huifen Wu, Huichun Pi, Yi Dong, Rong Xiang

**Affiliations:** ^1^Department of Nephrology, Xiangya Hospital, Central South University, Changsha, China; ^2^National Clinical Research Center for Geriatric Disorders, Xiangya Hospital, Central South University, Changsha, China; ^3^Department of Cell Biology, School of Life Sciences, Central South University, Changsha, China; ^4^Department of Medical Genetics and Prenatal Diagnosis, Shenzhen Longgang District Maternity and Child Healthcare Hospital, Shenzhen, China; ^5^Obstetric Inpatient Department, Shenzhen Longgang District Maternity and Child Healthcare Hospital, Shenzhen, China

**Keywords:** brain malformation, non-sense, mutation, NFIB, lung lobulation defects, lissencephaly

## Abstract

**Background:**

Nuclear factor I B (*NFIB*) plays an important role in regulating the transcription of multiple biological processes. Mutations in *NFIB* cause intellectual disability and macrocephaly. However, studies on abnormal brain and lung development caused by *NFIB* mutations are lacking.

**Methods:**

In the present study, we enrolled a fetus with brain malformation and lung lobulation defects from China. Whole-exome sequencing (WES) was performed to detect the candidate genes and Sanger sequencing was performed for mutational analysis.

**Results:**

After data filtering and bioinformatics prediction, a novel non-sense mutation of *NFIB* (NM_001190737:c.870C > A;p.Tyr290*) was identified in the fetus. This variant was predicted to produce a truncated NFIB protein because of a premature stop codon and was absent in 200 healthy controls.

**Conclusion:**

To the best of our knowledge, this is the first case of brain malformation and lung lobulation defects caused by a *NFIB* variant in Asia. These findings contribute to genetic diagnosis and family counseling and expand our understanding of *NFIB* mutations as well as brain and lung maturation.

## Introduction

The nuclear factor I (NFI) family was first described that took part in the replication of Adenovirus DNA (1). As a family of transcription factors, NFIs were later found to be required for the transcriptional regulation, particularly during development of each organ system (2). In humans and most vertebrates, there are four NFI family members: *NFIA*, *NFIB*, *NFIC*, and *NFIX*. All of them share a highly conserved DNA-binding and dimerization domain at their N-terminal (3).

As one of the important members of NFI family, NFIB can bind DNA and plays a critical role in the development of multiple organ systems (4), including the central nervous system (5–7), lungs (8), and mammary glands (9). Among those, NFIB is a site-specific DNA-binding protein that plays an important role in increasing chromatin accessibility and regulating the transcription of multiple biological processes. NFIB is closely related to different cancer types. An analysis of small cell lung cancer cells by Denny et al. revealed that high expression of Nfib altered the chromatin state globally to promote cancer metastasis (10). Moreover, Zilli et al. discovered that overexpression of *NFIB* was sufficient to enhance primary mammary tumor growth and promote lung metastatic colonization *via* increased *Ero1l*/*ERO1A* expression (11). Recent studies have shown that mutations in *NFIB* are associated with intellectual disability and macrocephaly (12–14). However, additional reports of *NFIB*-related cases remain lacking.

Here, we report an aborted fetus with brain malformation and lung lobulation defects and a novel non-sense mutation in *NFIB* (NM_001190737:c.870C > A;p.Tyr290*) using whole-exome sequencing (WES).

## Materials and Methods

### Subjects and Ethical Approval

The Review Board of Shenzhen Longgang District Maternity and Child Healthcare Hospital approved this study. The study was performed in accordance with the principles outlined in the Declaration of Helsinki in the ethics subsection. The parents provided written informed consent to participate in the study. Amniotic fluid was obtained from a mother undergoing amniocentesis. Blood was collected from the parents of the fetus.

### DNA Extraction and Exome Sequencing

Genomic DNA was extracted using a DNeasy Blood & Tissue Kit (Qiagen, Valencia, CA, United States). The main part of WES was performed at Guangdong Women’s and Children’s Hospital. The filtering strategies used conformed to those outlined in our previous study (15). Briefly, after preliminary quality control for the data, variants within intronic, intergenic and untranslated regions (UTRs), synonymous single nucleotide variants (SNVs), as well as variants with an alternative allele frequency more than 1% in the public databases [1000 Genomes, dbSNP144, YH database, and Genome Aggregation Database (gnomAD)], were firstly removed before further analysis. Then variants predicted by SIFT,^1^ PolyPhen-2,^2^ and MutationTaster^3^ as “Disease-causing,” were retained.

### Mutation Validation

Sanger sequencing was performed to confirm the potential causative variants in the family. Primer pairs were designed using the PrimerQuest Tool from IDT^4^; the PCR primer sequences are available upon request.

## Results

**FIGURE 1:**
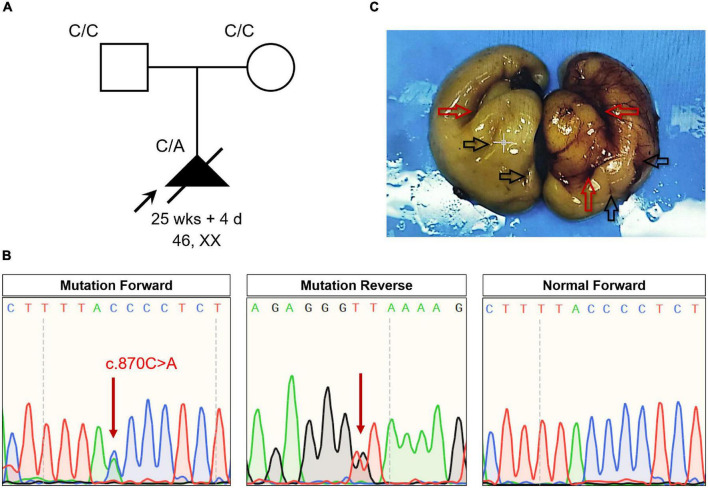
**(A)** The pedigree of this family. Black indicates the affected fetus with lissencephaly and lung lobulation defects. white circles/squares are unaffected. Arrow indicates the proband. **(B)** Sequencing results of the *NFIB* mutation. Sequence chromatograms indicate the heterozygosity of the *NFIB* non-sense mutation (NM_001190737:c.870C > A;p.Tyr290*) in the fetus and in a normal patient. Red arrow indicates the mutation. **(C)** Anatomical appearance of the cerebrum from the aborted fetus. Red arrows indicate the fissures of the brain. Black arrows indicate the brain sulci.

Increased nuchal translucency (NT) (4.8 mm) was detected in a fetus (13 weeks and 5 days) using ultrasonography (>2.5 mm could be considered as increased NT) (Figure 1A). Karyotyping and copy number variation analysis of amniotic fluid from the mother revealed no abnormalities (Supplementary Figure 1). Level III ultrasonography also showed no obvious abnormalities. To find the real cause of NT increased, the parents agreed to perform exome sequencing. Subsequent WES revealed a novel heterozygous non-sense mutation of *NFIB* (NM_001190737:c.870C > A;p.Tyr290*) in the fetus, which was verified by Sanger sequencing (Figure 1B). This mutation was absent in the parents. This novel non-sense mutation of *NFIB* (p.Tyr290*) resulted in a premature stop codon at position 290 in exon 6 of the *NFIB* gene; it was also absent in a local cohort comprising 200 control cases (15), and in the dbSNP, Exome Variant Server databases,^5^ and gnomAD. Meanwhile, all results from multiple bioinformatics software (MutationTaster, PolyPhen-2, and SIFT) indicated that the variant is “disease-causing.” According to the American College of Medical Genetics (ACMG) guidelines, the variant of *NFIB* (c.870C > A;p.Tyr290*) can be classified as Pathogenic, which meets the following criteria from the ACMG guidelines: PVS1 (null variant with evidence supporting for disease mechanism), PS2 (*de novo* variant), PM1 (functional domain variation), PM2 (absent from controls in the gnomAD), and PP3 (multiple lines of computational evidence showed the variant as “Disease-causing”).

Mutations in *NFIB* are known to be pathogenic for intellectual disability and macrocephaly, as well as pulmonary dysplasia in humans and mice (12). Therefore, the family decided to end their pregnancy by abortion at 25 weeks and 4 days. Anatomical findings showed that fetal body length and other developmental parameters were consistent with those at 26 weeks of gestation. The head circumference of the fetus was 220 mm (−2 to −3 SD); weight of cerebrum was 110 g, cerebellum was 8 g. However, there was no obvious structure of the gyrus and sulcus in the cerebral cortex (especially the left side of the brain) of the fetus (Figure 1C). Meanwhile, incomplete lobulation between the upper and middle lobes of the right lung was detected. Only a 1.0 cm of fissure was found on the right lung of the fetus. Abnormalities including biliary atresia and telecanthus (20 mm) were also observed in the fetus.

## Discussion

**FIGURE 2:**
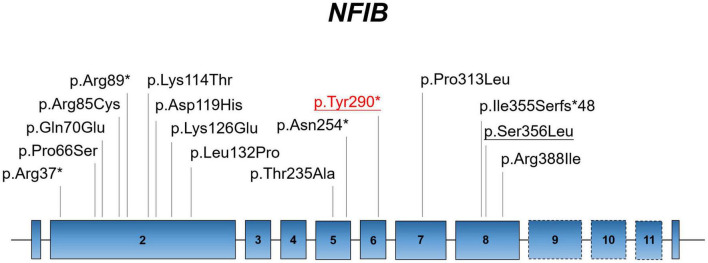
Localization of known mutations on the linear topology of *NFIB*. Red labeling represents the mutation identified in the present study. Underlined labels symbolize the fetal case.

The NFI family genes share a highly conserved DNA-binding domain; these genes regulate cell proliferation and differentiation of multiple organ systems. However, there are few studies on abnormal brain and lung development caused by *NFIB* deficiency. Currently, only 12 missense/non-sense and 2 small deletion/insertion variants of *NFIB* have been recorded in the Human Gene Mutation Database (HGMD; data retrieved in January 2022) (Figure 2). Here, we identified a novel non-sense *NFIB* variant (NM_001190737:c.870C > A;p.Tyr290*) in an aborted fetus. The variant was *de novo* (absent in parents) and resulted in premature termination by introducing a stop codon at the site of mutation. Our findings demonstrate the first case of abnormal brain development attributed to *NFIB* mutation (NM_001190737:c.870C > A;p.Tyr290*) in Asia, which expands our understanding of *NFIB* mutations and the associated physical development.

The case presented here manifested no obvious structure of the gyrus and sulcus in the cerebral cortex of the fetus, suggesting lissencephaly or brain developmental retardation (Figure 1C). Lissencephaly, which literally means “smooth brain,” was first described in 1914 (16, 17). Patients with lissencephaly also manifest intellectual disabilities, developmental delays, seizures, and muscle spasms (18, 19). In the present study, a novel non-sense *NFIB* variant (NM_001190737:c.870C > A;p.Tyr290*) was identified by using WES. Mutations in *NFIB* are known to cause disability and macrocephaly. However, lissencephaly is often accompanied by microcephaly instead of macrocephaly (20). Considering the fetus in our case was aborted at 25 weeks and 4 days, most of the gyri and sulci on the brain surface were still under development, the reason is more likely to be retardation in brain development caused by this *NFIB* mutation. Nevertheless, more in-depth evidences were needed to draw a definite conclusion.

Studies on *Nfib*-deficient mice have shown that loss of *Nfib* results in perinatal lethality due to lung maturation defects along with severe callosal agenesis and forebrain defects (21). Even *Nfib* hemizygous mice show reduced lung maturation and decreased survival (22, 23). Dorsal telencephalon-specific *Nfib* conditional knockout mice show an obvious increase in cortical size (12), which may explain the macrocephaly observed in *NFIB*-deficient patients. These results were partially consistent with our findings of brain malformation and right lung lobulation defects in a fetus, possibly as a result of a non-sense variant (p.Tyr290*) of *NFIB*. Our study is also the first report of lung maturation defects caused by *NFIB* deficiency in humans. Notably, the *NFIB* (c.870C > A;p.Tyr290*) variant detected in the present study has not been reported previously and is therefore a novel variant.

## Conclusion

We used WES to explore genetic lesions in a fetus with unexplained brain malformation and lung lobulation defects and identified a novel non-sense mutation of *NFIB* (NM_001190737:c.870C > A;p.Tyr290*). Our study thus contributes to insights for the genetic diagnosis and family counseling, and expands our understanding of *NFIB* mutations in association with brain and lung maturation.

## Data Availability Statement

The datasets for this article are not publicly available due to concerns regarding participant/patient anonymity. Requests to access the datasets should be directed to the corresponding author.

## Ethics Statement

The studies involving human participants were reviewed and approved by the Review Board of Shenzhen Longgang District Maternity and Child Healthcare Hospital. Written informed consent to participate in this study was provided by the participants’ legal guardian/next of kin.

## Author Contributions

RX conceived and directed the project. HH, LW, HW, and HP collected the data and information. HH, YD, and JJ analyzed and interpreted the data. HH and JJ wrote the manuscript with the help of all the other authors.

## Conflict of Interest

The authors declare that the research was conducted in the absence of any commercial or financial relationships that could be construed as a potential conflict of interest.

## Publisher’s Note

All claims expressed in this article are solely those of the authors and do not necessarily represent those of their affiliated organizations, or those of the publisher, the editors and the reviewers. Any product that may be evaluated in this article, or claim that may be made by its manufacturer, is not guaranteed or endorsed by the publisher.

## Footnotes

1
https://sift.bii.a-star.edu.sg

2
http://genetics.bwh.harvard.edu/pph2/index.shtml

3
http://www.mutationtaster.org

4
https://sg.idtdna.com/Primerquest/Home/Index

5
http://evs.gs.washington.edu/EVS/
