# Supplementary material for: A de novo Non-sense Nuclear Factor I B Mutation (p.Tyr290*) Is Responsible for Brain Malformation and Lung Lobulation Defects
Source: Front Pediatr. 2022 Mar 30;10:865181. doi: 10.3389/fped.2022.865181 (PMC9005976; doi:10.3389/fped.2022.865181)
Supplement: Supplementary file 1 [file Data_Sheet_1.DOCX]

Supplementary Material

# Supplementary Figure


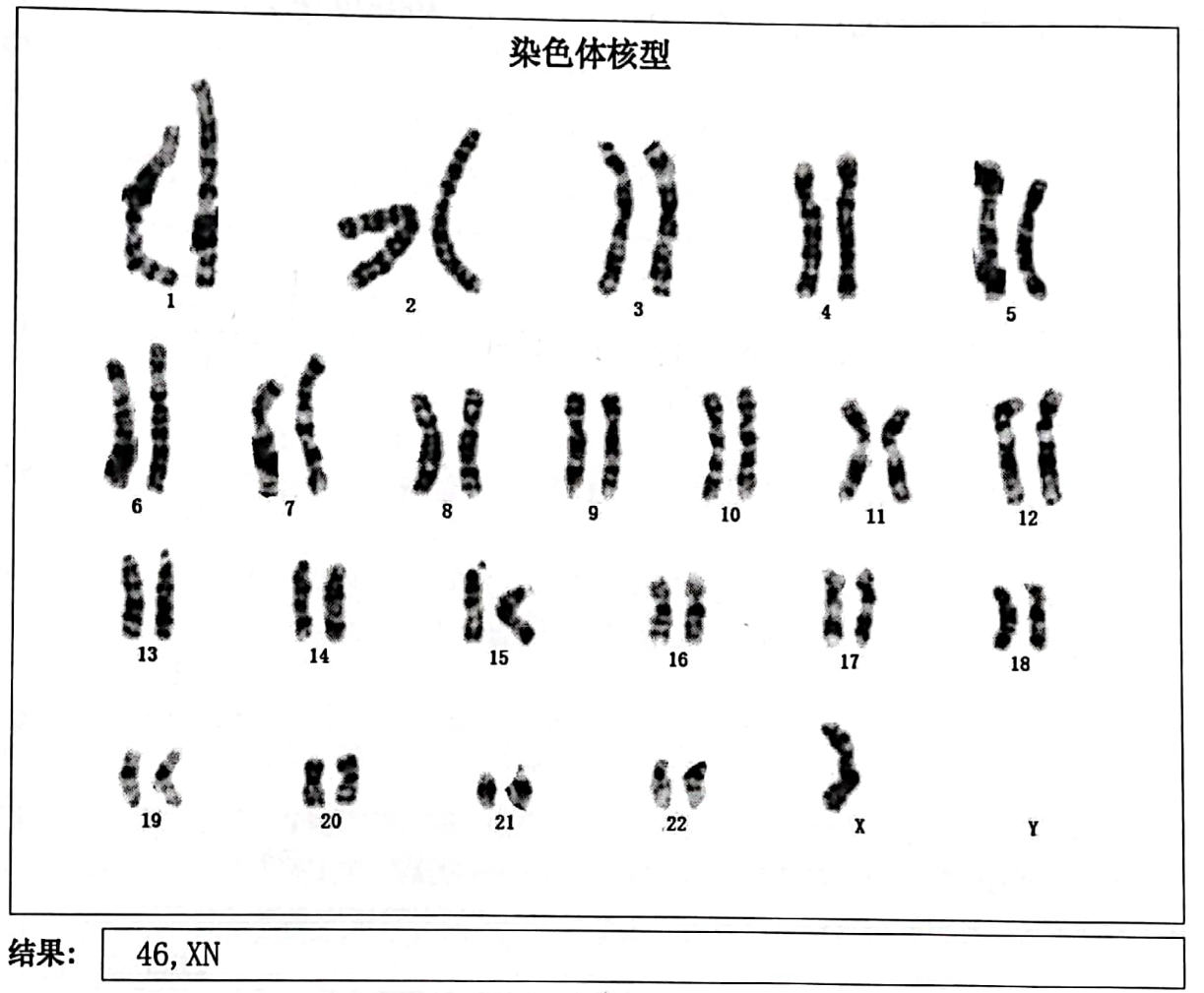


**Supplementary Figure 1.** Karyotyping analysis of amniotic fluid from the mother.
